# Candidate Molecular Biomarkers of Traumatic Brain Injury: A Systematic Review

**DOI:** 10.3390/biom14101283

**Published:** 2024-10-11

**Authors:** Tatiana V. Butkova, Kristina A. Malsagova, Valeriya I. Nakhod, Denis V. Petrovskiy, Alexander A. Izotov, Evgenii I. Balakin, Ksenia A. Yurku, Alexey S. Umnikov, Vasiliy I. Pustovoyt, Anna L. Kaysheva

**Affiliations:** 1 Institute of Biomedical Chemistry, 109028 Moscow, Russia; t.butkova@gmail.com (T.V.B.); vnakhod88@gmail.com (V.I.N.); petro2017@mail.ru (D.V.P.); izotov.alexander.ibmc@gmail.com (A.A.I.); kaysheva1@gmail.com (A.L.K.); 2 State Research Center—Burnasyan Federal Medical Biophysical Center, 123098 Moscow, Russiaks_yurku@mail.ru (K.A.Y.); dr.umnikov@yandex.ru (A.S.U.); vipust@yandex.com (V.I.P.)

**Keywords:** biomarkers, brain injury, metabolites, plasma, proteins, serum

## Abstract

Traumatic brain injury (TBI) is one of the leading causes of mortality and disability among young and middle-aged individuals. Adequate and timely diagnosis of primary brain injuries, as well as the prompt prevention and treatment of secondary injury mechanisms, significantly determine the potential for reducing mortality and severe disabling consequences. Therefore, it is crucial to have objective markers that indicate the severity of the injury. A number of molecular factors—proteins and metabolites—detected in the blood immediately after trauma and associated with the development and severity of TBI can serve in this role. TBI is a heterogeneous condition with respect to its etiology, clinical form, and genesis, being accompanied by brain cell damage and disruption of blood–brain barrier permeability. Two oppositely directed flows of substances and signals are observed: one is the flow of metabolites, proteins, and nucleic acids from damaged brain cells into the bloodstream through the damaged blood–brain barrier; the other is the infiltration of immune cells (neutrophils and macrophages) and serological proteins. Both flows aggravate brain tissue damage after TBI. Therefore, it is extremely important to study the key signaling events that regulate these flows and repair the damaged tissues, as well as to enhance the effectiveness of treatments for patients after TBI.

## 1. Introduction

Sequelae of traumatic brain injury (TBI) are complex and associated with a risk of developing negative delayed effects. Identification of candidate blood biomarkers to classify injury severity and outcome prognosis as well as assess the effectiveness of restorative measures are the important objectives in molecular neurobiology [1]. According to the national public health agency of the United States, Centers for Disease Control and Prevention (CDC), TBI is among the leading causes of mortality and disability worldwide; its severe sequelae can significantly affect quality of life of injured persons and their relatives. The main causes of nonfatal TBI include falls, bumps or collisions with objects, and motor vehicle accidents [2,3]. In 2019, in the US alone, the economic cost of injury was USD 4.2 trillion, including USD 327 billion in medical care, USD 69 billion in work loss, and USD 3.8 trillion in value of statistical life and quality of life losses. More than one half of this cost (USD 2.4 trillion) was among working-aged adults (aged 25–64 years) [4].

**Figure 1:**
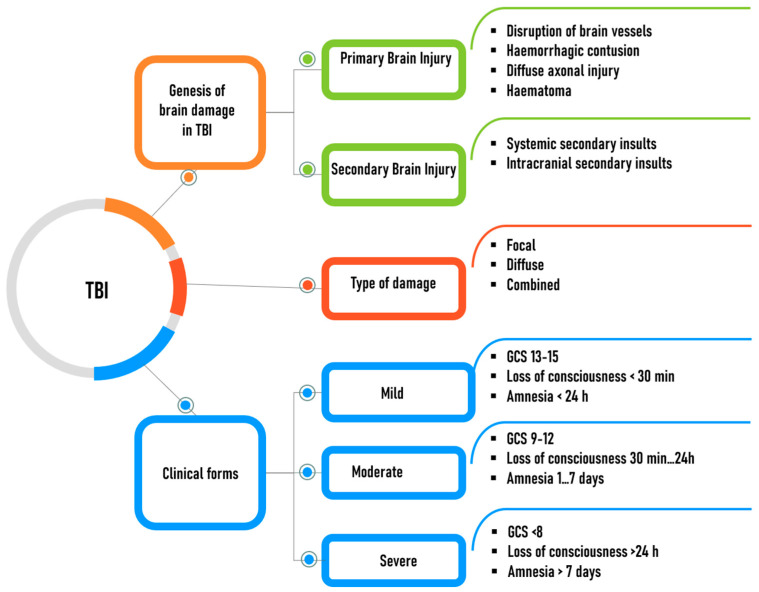
Classification of traumatic brain injury depending on type and genesis, clinical form, and severity of injury.

TBI progression is often accompanied by pathologic processes aggravating patient condition (the so-called secondary brain damage). Such processes involve activation of inflammation, apoptosis, excitotoxicity, or neuronal death [5,6]. Research into the molecular foundations of the pathogenesis helps understand how post-traumatic brain damage occurs and identify potential intervention points for developing new therapeutic options. TBI is a heterogeneous condition in terms of its genesis, clinical form, and course (Figure 1) [1].

The classification of TBI is rather complex (Figure 1). According to the 2022 report of the National Academies of Sciences, Engineering and Medicine, TBI can be subcategorized into mild (concussion), moderate, and severe clinical forms; however, the authors of the report believe that this classification needs to be revised [7]. The classification is based on the Glasgow Coma Scale (GCS), where a higher score indicates a milder form of TBI. The Russian national guidelines in neurology offer a classification of impaired consciousness proposed by A.N. Konovalov and T.A. Dobrokhotova, which is based on the GCS score. This classification comprises seven consciousness levels: fully conscious (awake); moderately and profoundly stunned; soporose; and moderately, deeply, and terminally comatose [8].

In terms of pathophysiology, brain damage in persons who have experienced TBI are categorized into primary and secondary injuries [9]. Primary injuries result from direct impact of traumatic factors: focal cerebral contusions, diffuse axonal injury, primary intracranial hematomas and their combinations. Secondary intracranial injuries result from such factors as increased BBB permeability, an increase in brain volume or swelling caused by edema, hyperemia or venous congestion, increased intracranial pressure, brain displacement and deformation, delayed hematomas (epi-/subdural or intracerebral), impaired circulation of blood and CSF as a result of subarachnoid or intraventricular hemorrhage, intracranial infection. Secondary extracranial factors include hypotension, hypoxemia, hypercapnia and anemia. The secondary factors can be prevented or cured, which depends on timely and correct diagnosis, as well as organization and quality of neurosurgical care rendered [10].

The pathoanatomic classification of a TBI describes the localization and/or morphology of the injury that is supposed to be treated. The pathoanatomic injuries can be categorized into focal, diffuse, or mixed ones [11,12,13]. In some publications, only two categories are singled out: focal and diffuse brain injuries [14,15].

Because of the complex nature of TBI at the molecular, cellular, and organ levels, it is difficult to predict its sequelae. The serious complications of TBI include paralysis and coma, as well as the high risk of mortality because of brain dysfunction. According to the statistics, the mortality rate among patients with meningeal damage is up to 70–80% [16]. The sequelae of TBI can be unpleasant even in the cases of mild trauma. Patients may experience mental disorders, memory impairment, as well as neurologic symptoms. They may also experience vision- or smell-related problems. These disorders can manifest themselves either immediately or several months after the injury (as delayed sequelae).

Motor vehicle accidents account for a significant share of TBIs. According to the WHO statistical report “World Health Statistics 2023: Monitoring health for the SDGs, Sustainable Development Goals” dated 19 May 2023, motor vehicle accidents were the leading cause of death among boys and young men aged 15–29 years in 2019 and the second most common cause of death among men aged 30–49 years [17].

Due to increased life expectancy, the WHO notes a shift in mortality patterns. Since the 2000s, there has been a steady rise in mortality caused by non-communicable diseases (NCDs) compared to infectious diseases. At the same time, the number of deaths related to injuries has remained constant at 8% of total deaths [17].

The high share of injury-related deaths emphasizes the relevance and urgency of diagnosing the injury status, including determining the severity of TBI. In order to reduce the burden of TBI, innovative treatments and interventions for persons who have experienced this type of injury need to be developed, and also special policies and programs need to be actively implemented for preventing injuries and reducing their frequency. These measures involve education and outreach initiatives, improvement of road and workplace safety, as well as promotion of innovative technologies, including early diagnosis, which can significantly reduce the risk of developing complications of TBI and improve treatment outcomes.

In this review, we analyzed the available literature in order to describe candidate molecular factors: proteins and metabolites, which are detected in blood immediately after injury and are potentially associated with TBI development and severity of its course. In addition to a clear classification of traumatic brain injury (TBI) based on biomechanical characteristics, type, nature, form, severity and clinical presentation, researchers should also consider other factors such as gender, age and time, which affect changes in the composition and concentration of the biomolecules in probes [18].

The focus of the research has shifted toward closed head injuries, which do not involve disruption of the integrity of the soft tissues of the head and/or aponeurotic stretching of the skull, as well as secondary damage, in order to avoid analysis of the brain and meninges infection effects.

## 2. Methodology

**Figure 2:**
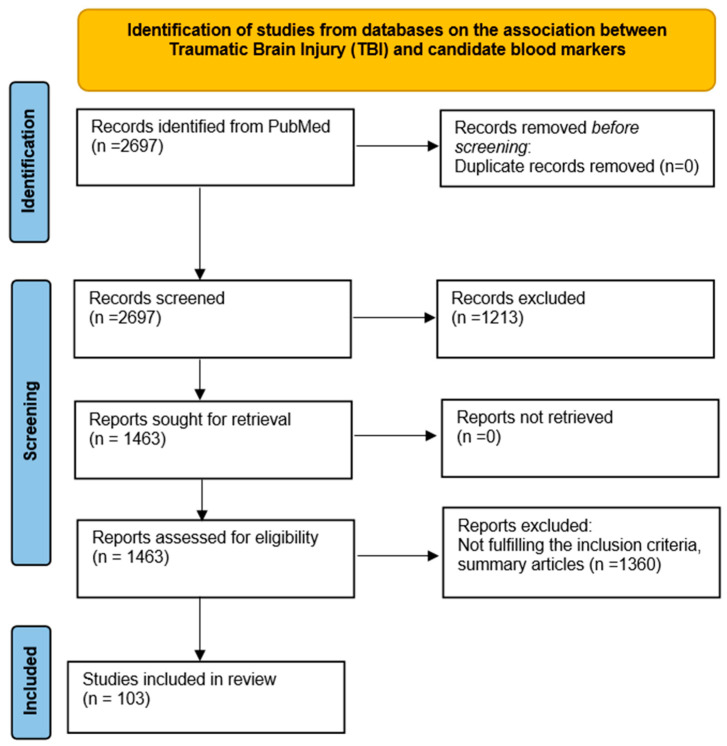
PRISMA flow diagram for the review.

This study is conducted following the PRISMA statement and the Review was not registered. For conducting this review, we performed an extensive literature search in the PubMed database to identify relevant publications. The review authors held collaborative discussions to consolidate their findings and prepare the current narrative review (Figure 2).

Inclusion Criteria: We applied several inclusion criteria to select studies. Only full-text articles in English that presented case reports, case series, or reviews involving patients diagnosed with TBI and the presence of molecular factors—proteins and metabolites—detected in the blood immediately after the injury were included. Only articles containing relevant data were considered.

Exclusion Criteria: The exclusion criteria included articles published in languages other than English, general reviews, articles with insufficient data, articles without full-text access, and studies involving other types of injuries without detailed investigation of TBI. We note a dearth of descriptions regarding the nature of the injuries across the analyzed studies. This deficiency primarily relates to the assessment of the damage severity.

All identified articles were analyzed and screened. The evaluated articles were published between May 2014 and May 2024. A total of 2697 articles were identified using the search criteria. Subsequently, 2573 articles were excluded due to a lack of relevant data (full text, publication date within the last 10 years, species: human, age: adults, classical article, clinical study, clinical trial, meta-analysis, review, systematic review). The remaining 103 articles were further assessed for eligibility and selected for the study.

## 3. Candidate Blood Biomarkers of TBI

Clinical analysis methods play a crucial role in understanding the molecular mechanisms underlying secondary injury and the stages of pathological neuroprocesses, particularly through cerebral microdialysis (CMD). CMD is a laboratory approach based on in vivo membrane sampling for the subsequent monitoring of changes in the concentrations of endogenous metabolites and pharmaceuticals within brain tissue [19]. Currently, studies have provided insights into the in vivo monitoring of cerebral physiology following traumatic brain injury of varying severity. Evaluation of the dynamics of cerebral metabolic dysfunction after traumatic brain injury of varying severity and analysis of the content of endogenous metabolites glucose, glutamate, lactate/pyruvate ratio, etc. [20]. Of particular interest is the development of CMD towards the identification of proteinaceous biomarkers in microdialysate using high-sensitivity quantitative methods like HPLC-MS/MS, as well as the study of blood–brain barrier permeability utilizing isotopes of low molecular weight compounds in conjunction with HPLC-MS/MS analysis [21].

The secondary injury mechanisms are triggered at the instant of a traumatic event, are accompanied by a number of molecular, cellular, and physiological processes affecting all types of brain cells, and can last for a long period (ranging from several months to several years) [22]. Blood–brain barrier (BBB) dysfunction, excitotoxicity, mitochondrial dysfunction, oxidative stress, and inflammation are the key mechanisms of secondary brain injury [23].

TBI is accompanied by significant changes in expression and activation of various proteins playing a crucial role in the pathophysiology of this condition. Some of these proteins include pro-inflammatory factors, stress proteins, growth factors, and pro-apoptotic proteins. Pro-inflammatory factors such as cytokines (e.g., interleukins and tumor necrosis factor) are activated in response to injury and can enhance the inflammatory response, thus contributing to further tissue damage. Stress proteins, such as heat shock proteins, respond to injury and help cells adapt to stressful conditions, but their overactivation can also exacerbate the pathologic processes. Growth factors such as neurotrophic factor (NTF) stimulate neuroregeneration and survival as well as help recover after injury. Pro-apoptotic proteins mediate programmed cell death and may play a crucial role in determining the fate of damaged cells.

**Figure 3:**
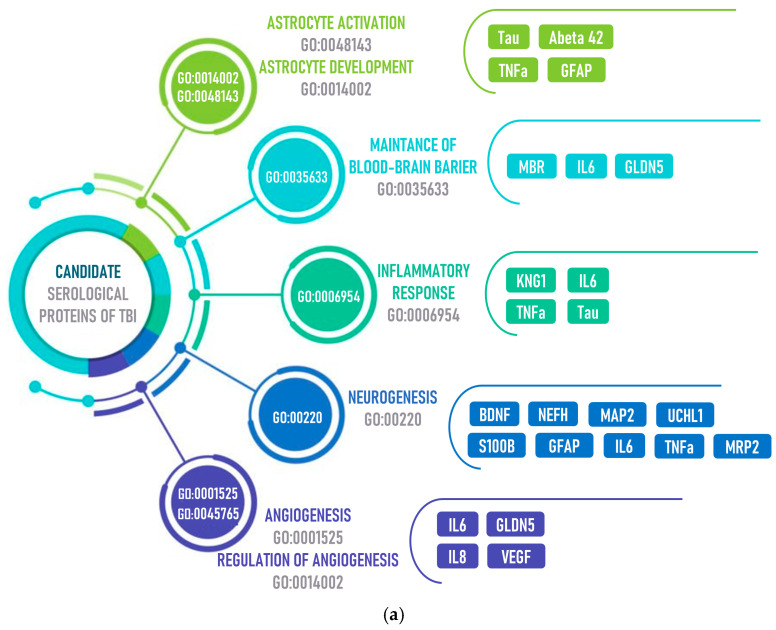

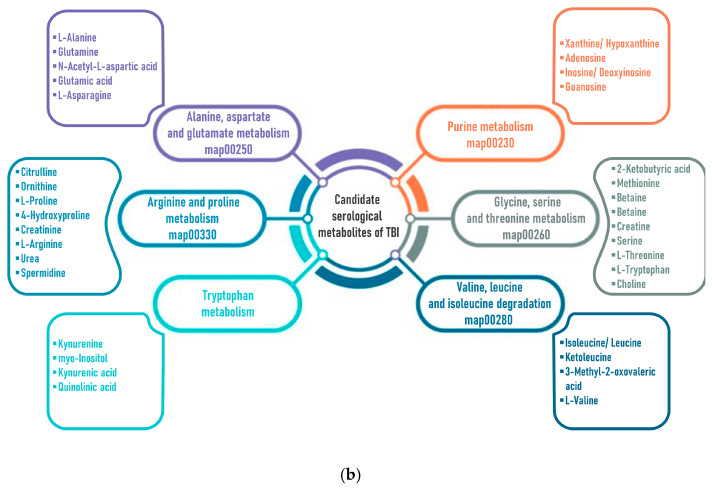
The processes and molecular factors involved in them: proteins (**a**) and metabolites (**b**) identified in blood after TBI.

In addition to implementation of the aforementioned defense mechanisms, which often aggravate the pathological condition, proteins, nucleic acids, metabolites are released from neurons and glia into the extracellular space and further into the bloodstream due to increased BBB permeability and glymphatic clearance (Figure 3).

Figure 3 shows proteins and metabolites participating in the pathophysiological mechanisms of TBI. Figure 3a demonstrates the major processes in which proteins are involved. These processes include astrocyte activation and development. The levels of tau-protein, tumor necrosis factor (TNFα), and glial fibrillary acidic protein (GFAP) are increased; they play an essential role in regulation of the inflammatory and remodeling processes in the brain, as well as restoration of tissues and functions after injury. TBI leads to neuroinflammation, neurodegeneration, increased permeability of the blood–brain barrier, and microvascular damage to the brain. Inflammatory mediators secreted by activated glial cells, neurons, and mast cells contribute to the pathogenesis of TBI through secondary brain damage [24].

TBI is followed by a rapid inflammatory and immune response involving astrocyte activation. Astrocytes become hypertrophied and start producing a variety of bioactive molecules including cytokines, chemokines, growth factors, and stress proteins [25]. These molecules can exhibit both protective and detrimental effects on the surrounding tissues and neurons. Astrocyte activation is also accompanied by morphological and functional changes. Activated astrocytes are involved in scar tissue formation and limit the spread of brain damage area. Furthermore, astrocytes may play a crucial role in tissue repair and remodeling after injury through glial scar formation and secretion of growth factors. Along with activation, TBI may also affect astrocyte development. This process may involve accelerated development of certain astrocyte subtypes, as well as alteration in expression of the genes associated with their differentiation and function. [26].

In recent years, our understanding of the functional role of astroglia has evolved. Previously, these cells were mainly viewed as supporting the structure and metabolism of the brain. However, it is now established that they actively influence synaptic processes by modulating the effects of neurotransmitters. Through their interactions with neurons, astrocytes impact the formation of neural networks and play a role in regulating memory, as well as complex behavioral responses, both under normal conditions and in the context of cerebral pathologies [27]. It has been demonstrated that reactive A1 astrocytes can induce neuronal death after traumatic brain injury through the secretion of the neurotoxic complement component C3 [28].

Besides the key role in the reactions to central nervous system injuries through processes known as reactive astrogliosis, astrocytes play a critical role in the early response to brain damage [29]. Pharmacological targeting of astrocytes appears to be the most promising therapeutic strategy for treating the consequences of TBI. In addition to synthesizing proteins and neurotrophic factors, astrocytes play a crucial role in activating gliosis and forming glial scars, which can have both positive and negative effects on the development of pathological processes associated with TBI. On one hand, the glial scar isolates the damaged tissue from healthy tissue, preventing further spread of the injury. On the other hand, it hinders axon growth and the regeneration of synaptic connections, which limits functional recovery [30].

Some proteins are involved in maintaining the structural and functional integrity of the blood–brain barrier after TBI: MBP (mannan-binding protein), IL6 (interleukin 6), and CLDN5 (claudin-5). Their changes in response to injury may affect BBB permeability and have important implications for the pathophysiology and treatment used [31,32].

The inflammatory response involving interleukins also plays a crucial role in the pathophysiology of traumatic brain injury. In the early phase, activation of inflammatory processes may be a defense mechanism aiming to activate microglia and other immune cells involved in phagocytosis. However, if the inflammatory response is excessive and persists for a long time, the process can become pathologic and lead to additional tissue damage. Therefore, understanding the balance between the protective and pathologic sequelae of the inflammatory response is important for elucidating treatment and rehabilitation strategies for patients with TBI [33,34].

Neurogenesis is another important aspect of tissue regeneration and repair after TBI. It is noteworthy that when microglia and astroglia are overactive, expression of cytokines (TNF or tumor necrosis factor, interleukin 1) and chemokines is upregulated [35]. Although the ultimate goals of glial cell-mediated neuroinflammation are threat elimination, formation of a glial scar, resolution of the resulting damage, and restoration of brain homeostasis, prolonged exposure of macrophages to inflammatory stimuli can induce neurotoxic reactive astrocytes [36,37].

Loss of vascular integrity plays a pivotal role in mediated tissue damage after TBI. Destruction of microvascular walls of the blood–brain barrier activates the coagulation cascade leading to ischemia in the areas surrounding the primary injury site, followed by reduction of cerebral blood flow velocity (the “steal” syndrome). All these factors demonstrate that it is important to restore blood supply and perform vascular remodeling in the damaged brain area involving CLDN5 (claudin 5), VEGF (vascular endothelial growth factor), and other proteins [38,39].

Changes in the metabolome in response to pathology development are also important for understanding the pathogenesis. The levels of endogenous metabolites are very sensitive to changes in the body state (Figure 3b).

Brain damage results in activation of neuronal metabolism, accompanied by ATP depletion and impaired calcium pump function [40]. Purine bases are released from tissues under conditions of metabolic stress and lack of mitochondrial substrates (e.g., in patients with cardiac or cerebral ischemia) or due to ATP deficiency.

Glycine is the major inhibitory neurotransmitter in the brainstem and spinal cord; it is metabolized from L-serine, which binds to glycine receptors. Serine exerts a neuroprotective role in the CNS by reducing neurotoxicity through activation of glycine receptors and affects the inflammatory response by decreasing the levels of pro-inflammatory factors. Hence, reduced serine levels may intensify inflammatory responses and cerebral ischemia after traumatic brain injury [41].

Biosynthesis impairment and degradation of valine, leucine, and isoleucine emphasize the role played by branched-chain amino acids (BCAAs) in the pathology of TBI. In the brain, BCAAs act as important metabolic precursors required for the synthesis of proteins and neurotransmitters such as dopamine, serotonin, and norepinephrine. Being an important source of nitrogen, BCAAs also contribute to the synthesis of major brain metabolites: glutamate and glutamine. Furthermore, BCAAs was shown to play a crucial role in recovery after TBI. Therefore, reduction of the level of these amino acids is directly proportional to TBI severity [41,42].

Tryptophan is vital for the synthesis of all proteins and essential for cells of the peripheral and central nervous systems; it affects the immune system function. Reduced tryptophan levels inhibit the proliferation of peripheral nuclear cells and activation of heterologous immune cells, as well as increase the suppression of T-cell response. TBI causes persistently abnormal tryptophan metabolism, with transient shifts in different pathways of tryptophan metabolism. These changes are potentially associated with the acute and chronic TBI trajectory, although the exact mechanism remains unclear. Therapeutic interventions targeting tryptophan metabolism may affect the prognosis of TBI and discover new treatment options [43].

Arginine and proline metabolism can also be impaired after traumatic brain injury. These amino acids play essential roles in the metabolic and immune processes associated with recovery after injury and neural tissue protection. Disruption of the arginine and proline metabolism may cause an imbalance in synthesis of nitric oxide, as well as impair creatine and collagen synthesis, which can have a negative impact on restoration of the function of nervous tissue [44].

The metabolism of alanine, aspartate and glutamate plays a crucial role in neurometabolism and amino acid metabolism. Thus, excess level of glutamate after a traumatic brain injury leads to its excitotoxicity [41,45].

### 3.1. Changes in the Blood Levels of Candidate Proteins after TBI

Proteins are key components in the pathophysiology and subsequent recovery after TBI. Studying proteins can help understand the mechanisms of injury, develop diagnostic and therapeutic approaches, and assess the outcome prognosis after injury. Emergence of protein markers in the bloodstream is characteristic of each degree of TBI severity (Table 1).

Identification of protein markers of brain damage in human biological fluids is an important method for assessing the degree of neuronal damage and an informative indicator of neurological deficit in patients with traumatic brain injury. These markers can provide valuable information on injury severity and the outcome prognosis, which allows one to early assess the pathologic condition in patients.

### 3.2. Changes in the Blood Levels of Endogenous Metabolites after TBI

Studying metabolites in biological matrices is a relevant approach for identifying biomarkers and developing personalized treatment regimens [97]. Metabolomics has significant research potential regarding pathophysiological processes in patients who have experienced a traumatic brain injury, as it enables detection of systemic changes and the involved metabolic pathways (Table 2).

The metabolomic biomarkers listed in Table 2 participate in different biochemical reactions and biological pathways that accompany TBI and may help to build a picture of the evolving pathologic process. Thus, a reduced level of tauroursodeoxycholic acid, which is associated with apoptosis prevention and mitochondrial protection, explains the cellular and mitochondrial damage processes observed in patients after TBI at the molecular level [101]. The level of the oxidative stress marker, methionine sulfoxide, increasing with time after TBI explains some symptoms of secondary brain damage [107,108]. Candidate biomarkers (Table 2) are mainly involved in amino acid, lipid, and carbohydrate metabolism as well as the tricarboxylic acid cycle. Changes in their levels after TBI can presumably be associated with metabolic dysregulation [109,110].

## 4. The Key Mechanisms of Brain Damage

BBB impairment is an early event that occurs within several hours after injury [6,111]. Primary brain damage caused by a forceful blow, injury to the dura mater and parenchyma result in TBI of different severity [112]. Secondary damage then develops within several hours or days, being mediated by impaired transport of potassium, sodium, and calcium cations as well as an increase in the level of reactive oxygen species (ROS). Early detection of secondary brain damage is a challenge in molecular pathophysiology of TBI. Secondary brain damage is accompanied by excitotoxicity, oxidative stress, inflammation, and apoptosis.

**Figure 4:**
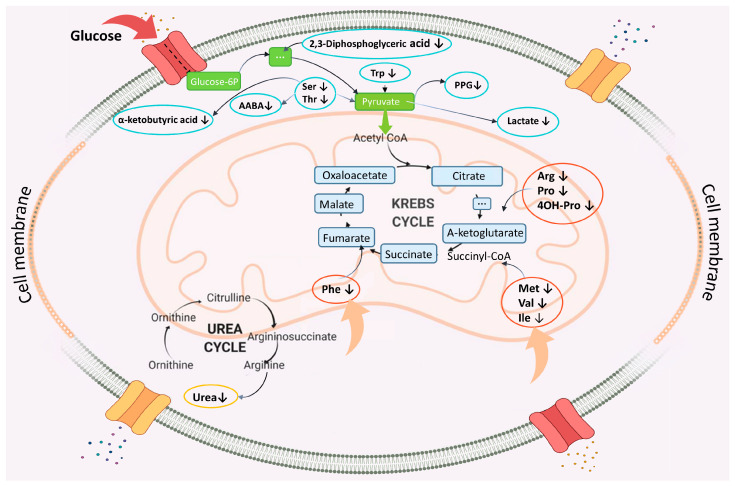
Metabolic disorders in patients after TBI. Arrows show the reduced blood levels of proteins or endogenous metabolites associated with the development of TBI. TCA—the tricarboxylic acid cycle (the Krebs cycle); AABA—α-aminobutyric acid; PG—propylene glycol.

Excitotoxicity is a pathological process accompanied by release of neurotransmitters and neuronal death [5]. The following mechanisms of this process are currently known: NMDAR (N-methyl-d-aspartate receptor)-dependent excitotoxicity, mechanoporation, and GABA (gamma-aminobutyric acid)-mediated excitotoxicity [5]. NMDAR receptors are glutamate receptor ion channels that are important mediators of excitatory neurotransmission and synaptic plasticity [113]. Glutamate, a major excitatory neurotransmitter, is essential for normal brain function. However, metabolic disturbances occurring immediately after neurotrauma cause profound mitochondrial dysfunction and loss of ATP production (Figure 4) [114,115].

Figure 4 shows that mitochondrial dysfunction can be accompanied by reduced levels of the key endogenous metabolites participating in glycolysis, the tricarboxylic acid cycle, as well as the metabolism of canonical amino acids and carboxylic acids, and the urea cycle. ATP production and the ATP/ADP ratio decrease; diphosphate accumulation in mitochondria occurs. Further cerebral metabolism imbalance develops. Energy demand cannot be met by the universal ATP “currency” being formed, which eventually leads to destruction of ATP-dependent Ca^2+^ pumps, changes in local levels of creatine, creatine phosphate and N-acetyl aspartate [116]. Acute excitotoxicity is mediated by depolarization of the postsynaptic membrane and osmotic imbalance. In this case, a steady Ca^2+^ influx through glutamate receptor channels is a common pathway of neuronal cell death [23].

Oxidative stress is another key process in neuropathology. The association between oxidative stress and neuronal death has been widely reported in the literature. Development of oxidative stress exacerbates mitochondrial dysfunction and energy deficiency, leading to the release of pro-apoptotic factors. Increased ROS levels also causes neuronal cell death and release of intracellular metabolites into the extracellular space [117].

Traumatic brain injury results in release of biomolecules into the extracellular space and further into the bloodstream. It is the release of proteins and metabolites in blood from brain cells that is attractive as a molecular marker of the severity of disease progression. Thus, astrocytes, neuroglial cells with an extremely diverse morphology and functions are the source of S100B and GFAP; these cells are involved in many physiological processes such as regulation of axonal growth and support, blood–brain barrier formation, and immune responses [118]. UCH-L1, Tau, and NF-L proteins appear in the bloodstream as a result of neuronal death. On the other hand, an opposite process is observed: infiltration by immune cells (neutrophils and macrophages) and serologic proteins, aggravating the inflammatory response [119]. The blood levels of kininogen-1 (KNG-1), interleukins (IL6 and IL8), and TNFα increase simultaneously.

## 5. Conclusions

Traumatic brain injury (TBI) is increasingly being recognized as a global health public priority because most injuries can be prevented, while long-term and expensive medical care is needed in the case of moderate and severe brain injuries. TBI-related mortality disproportionately affects young men of working age. Early diagnosis of TBI is critical for ensuring optimal outcomes for individuals who have experienced TBI, as it helps prevent serious complications and improves prognosis. Despite the large body of academic research that focuses on searching for and characterizing candidate molecular factors (proteins and metabolites in the blood of patients with TBI, which are possibly associated with the development of this disease), there is still a lack of approved biomarkers for traumatic brain injury to be used in clinical practice for both adults and children.

The development of omics technologies aimed at identifying molecular features associated with disease progression is currently a priority. The feasibility of performing personalized biomarker monitoring in patients after traumatic brain injury will improve the effectiveness of therapeutic measures and surgical interventions.

## Figures and Tables

**Table 1:** Candidate blood protein markers that are promising for diagnosing traumatic brain injury.

Candidate Biomarkers	Event	Change	Diagnosis/Prognosis	Pathogenesis	Biosample	Participants	Method Detection	Limit of Detection	Refs.
Tau	Astrocyte activation GO:0048143 Astrocyte development GO:0014002 Inflammatory response GO:0006954	↑	Marker of moderate/severe TBI (AUC 0.63)	oxidative stress	blood	122	bead-based immunoassay	–	[46,47,48,49]
Abeta 42	Astrocyte activation GO:0048143 Astrocyte development GO:0014002	↑	Marker of TBI, predictor of neurological status and mortality after TBI	oxidative stress	plasma	12	immunoassay	0.02 ng/L	[50,51]
TNFa	Astrocyte activation GO:0048143 Astrocyte development GO:0014002 Inflammatory response GO:0006954	↑	Marker of TBI, predictor of chronic phases	oxidative stress	plasma	160	electrochemiluminescent assay	0.04 ng/L	[28,52,53,54,55,56]
GFAP	Astrocyte activation GO:0048143 Astrocyte development GO:0014002 Neurogenesis GO:00220	↑	prediction of mortality after TBI (AUC 0.84 ± 0.05, <12 h)	neurogenesis synaptogenesis	serum	92	immunoluminometric assay	0.03 μg/L	[28,57,58,59,60,61,62,63,64]
TLR	Astrocyte activation GO:0048143	↑	therapeutic targets for cerebrovascular disorders	inflammatory response	blood	146	flow cytometry	–	[60,65]
SUR1	Astrocyte activation GO:0048143	↑	Prognose of severity, poor outcome after hemorrhagic stroke (~0.8 AUC)	–	serum	262	immunoassay	<1 μg/L	[66,67]
BDNF	Astrocyte activation GO:0048143 Neurogenesis GO:00220	↑	Marker of severe TBI, predictor of mortality after TBI	neurogenesis	plasma	120	immunoassay	16 ng/L	[57,60,68,69,70]
MBP	Maintenance of blood–brain barrier GO:0035633	↑	Marker of severe TBI, predictor of mortality after TBI (AUC 0.83)	–	plasma	127	–	–	[1,57,71,72]
IL6	Maintenance of blood–brain barrier GO:0035633 Inflammatory response GO:0006954 Angiogenesis GO:0001525	↑	Clinical Diagnosis and Severity (AUC 0.92)	inflammatory response	plasma	160	electrochemiluminescent assay	0.06 ng/L	[28,53,55,56,61,71,73,74,75,76]
CLDN5	Maintenance of blood–brain barrier GO:0035633	↓	Marker of chronic mild TBI	inflammatory response	plasma	84	fluorescent immunoassay	–	[38,39,77]
IL8	Inflammatory response GO:0006954 Angiogenesis GO:0001525	↑	Clinical Diagnosis and Severity (AUC 0.76)	inflammatory response	plasma	160	electrochemiluminescent assay	0.07 ng/L	[28,53,55,56,74,75,76]
CCL2	Inflammatory response GO:0006954	↑	Marker of TBI (AUC > 0.62)	inflammatory response	plasma	130	fluorescent immunoassay	~60 ng/L	[74,78,79]
IL-10	Inflammatory response GO:0006954	↓	Clinical Diagnosis and Severity (AUC 0.86)	inflammatory response	plasma	160	electrochemiluminescent assay	0.04 ng/L	[55,56,75,76,80,81]
NEFH	Neurogenesis GO:00220	↑	Marker of mild TBI (AUC 0.83)	neurogenesis	plasma	42	MRM-MS	–	[82,83,84,85]
MAP2	Neurogenesis GO:00220	↓	Prognosis of severe TBI	neurogenesis	serum	32	immunoassay	<0.01 μg/L	[59,86,87,88]
UCHL1	Neurogenesis GO:00220	↑	Marker of moderate/severe TBI (AUC 0.61)	neurogenesis	blood	122	bead-based immunoassay	1.90 ng/L	[49,57,58,89]
S100B	Neurogenesis GO:00220	↑	prediction of mortality after TBI, brain injury marker of circulatory arrest, st, TBI (AUC 0.82 ± 0.06, 85–108 h)	neurogenesissynaptogenesis	serum	92	immunoluminometric assay	0.02 μg/L	[62,90,91]
NRF2	Neurogenesis GO:00220	↑	Marker of TBI, prognose of early neurologic deterioration (AUC 0.77–0.8, 6–24 h)	neurogenesis	serum	230	immunoassay	–	[92]
VEGF	Regulation of angiogenesis GO:0045765	↑	Marker of mild TBI (AUC 0.86, 24 h)	angiogenesis	plasma	250	immunoassay	–	[93,94,95,96]

**Table 2:** Promising endogenous metabolites for the diagnosis of traumatic brain injury.

Metabolite	Pathway Name	Pathway ID	Change	Blood	Refs.
L-alpha-Aminobutyric acid	Cysteine and methionine metabolism	map00270	↓	Serum	[98]
Methionine sulfoxide			↑	Serum	[99]
3-Hydroxybutyric acid	Glycolysis/Gluconeogenesis	map00010	↑	Serum	[99]
2,3-Diphosphoglyceric acid			↓	Plasma	[100]
Glycerol	Pentose and glucuronate interconversions	map00040	↑	Serum	[99,101]
Caprylic acid	Fatty acid biosynthesis	map00061	↑	Serum	[83,85,86]
Capric acid			↓	Plasma	
Glycine	Primary bile acid biosynthesis	map00120	↑	Plasma	[100]
Thymine	Pyrimidine metabolism	map00240	↓	Serum	[99,101]
Glutamic acid	Alanine, aspartate and glutamate metabolism	map00250	↑	Plasma	[45]
L-Asparagine			↑	Plasma	[102]
2-Ketobutyric acid	Glycine, serine and threonine metabolism	map00260	↓	Plasma	[102]
Methionine			↓	Plasma	[102]
Betaine			↑	Plasma	[103]
Creatine			↓	Serum	[99,100,101]
Serine			↓	Serum	[101]
L-Threonine			↓	Plasma	[104]
L-Tryptophan			↓ and ↑	Plasma	[100,102]
Choline			↓	Plasma	[102]
Dimethylglycine			↓	Plasma	[104,105]
Isoleucine	Valine, leucine and isoleucine degradation	map00280	↓	Plasma	[105]
Ketoleucine			↓	Plasma	[104,105]
3-Methyl-2-oxovaleric acid			↓	Plasma	[104,105]
L-Valine			↓	Plasma	[104,105]
Leucine			↓	Plasma	[105]
Isoleucine			↓	Plasma	[100,103]
Citrulline	Arginine and proline metabolism	map00330	↓	Plasma	[103]
Ornithine			↓	Plasma	[100,102,103]
L-Proline			↓	Plasma	[103]
4-Hydroxyproline			↓	Plasma	[103]
Creatinine			↓	Plasma	[103]
L-Arginine			↓	Plasma	[87]
Urea			↓	Serum	[106]
Proline			↑	Serum	[100,101]
Kynurenic acid	Tryptophan metabolism	map00380	↑	Plasma	[104]
Quinolinic acid			↑	Plasma	[104]
Phenylalanine	Phenylalanine, tyrosine and tryptophan biosynthesis	map00400	↓	Plasma	[102]
L-Tyrosine			↑	Plasma/Serum	[99,102]
2-Hydroxybutyric acid	Propanoate metabolism	map00640	↑	Plasma	[100]
Lactic acid	Butanoate metabolism	map00650	↓	Plasma	[100]
Niacinamide	Nicotinate and nicotinamide metabolism	map00760	↓	Plasma	[105]
Glucose 6-phosphate	Insulin secretion	map04911	↓	Plasma	[105]
Isobutyryl-L-carnitine	n/d*	n/d	↓	Plasma	[105]
Propionylcarnitine	n/d	n/d	↓	Plasma	[105]
Succinylcarnitine	n/d	n/d	↓	Plasma	[105]
2-Hydroxyisovalerylcarnitine	n/d	n/d	↓	Plasma	[105]
2-Methylbutyroylcarnitine	n/d	n/d	↑	Plasma	[105]
Isovalerylcarnitine	n/d	n/d	↓	Plasma	[102]
Methylglutarylcarnitine	n/d	n/d	↓	Plasma	[102]
Cysteine hydrochloride	n/d	n/d	↓	Plasma	[102]
gamma-Glutamylvaline	n/d	n/d	↓	Plasma	[102]
gamma-Glutamylleucine	n/d	n/d	↓	Plasma	[102]
gamma-Glutamylisoleucine	n/d	n/d	↓	Plasma	[102]
gamma-Glutamyltyrosine	n/d	n/d	↑	Serum	[98]
epi-Inositol	n/d	n/d	↑	Serum	[98]
Propylene glycol	n/d	n/d	↓	Serum	[99]
Ribonic acid	n/d	n/d	↓	Plasma	[105]
Indole-3-propionic acid	n/d	n/d	↓	Plasma	[105]
2-methylbutyrylcarnitine	n/d	n/d	↓	Plasma	[100]
Isobutyryl-L-carnitine	n/d	n/d	↓	Plasma	[101]

*—no data.

## Data Availability

This is a review paper that collected from public data listed in the “References” and from open access web-source PubMed.
